# Mechanisms underlying heterologous skin scaffold-mediated tissue remodeling

**DOI:** 10.1038/srep35074

**Published:** 2016-10-11

**Authors:** Kallyne K. O. Mimura, Andréia R. Moraes, Aline C. Miranda, Rebecca Greco, Tahera Ansari, Paul Sibbons, Karin V. Greco, Sonia M. Oliani

**Affiliations:** 1 Post-Graduation in Structural and Functional Biology, Federal University of São Paulo (UNIFESP), São Paulo, SP, 04023-900, Brazil; 2 Department of Biology; Instituto de Biociências, Letras e Ciências Exatas; São Paulo State University (UNESP), São José do Rio Preto, SP, 15054-000, Brazil; 3 Department of Surgical Research, Northwick Park Institute for Medical Research, University College London (UCL), London, Middlesex, HA1 3UJ, United Kingdom

## Abstract

Biocompatibility of two newly developed porcine skin scaffolds was assessed after 3, 14, 21 and 90 days of implantation in rats. Both scaffolds showed absence of cells, preservation of ECM and mechanical properties comparable to non-decellularised skin before implantation. Host cell infiltration was much prominent on both scaffolds when compared to Permacol (surgical control). At day 3, the grafts were surrounded by polymorphonuclear cells, which were replaced by a notable number of IL-6-positive cells at day 14. Simultaneously, the number of pro-inflammatory M1-macrophage was enhanced. Interestingly, a predominant pro-remodeling M2 response, with newly formed vessels, myofibroblasts activation and a shift on the type of collagen expression was sequentially delayed (around 21 days). The gene expression of some trophic factors involved in tissue remodeling was congruent with the cellular events. Our findings suggested that the responsiveness of macrophages after non-crosslinked skin scaffolds implantation seemed to intimately affect various cell responses and molecular events; and this range of mutually reinforcing actions was predictive of a positive tissue remodeling that was essential for the long-standing success of the implants. Furthermore, our study indicates that non-crosslinked biologic scaffold implantation is biocompatible to the host tissue and somehow underlying molecular events involved in tissue repair.

Designed as a skin substitute, both epidermal and dermal acellular matrices (scaffolds) obtained after different decellularisation processes have been under investigation for over a decade^1^,^2^,^3^,^4^,^5^.

The choice of an appropriate scaffold is important to guide cell behavior, and cytotoxic products or materials that induce extensive scar formation should be avoided^6^. Differences in manufacturing techniques such as decellularization and crosslinking protocols can alter the physical characteristics of scaffolds^7^. To resist forces like wound contraction, scaffold materials - such as Permacol - are chemically crosslinked to enhance strength. However, because of lower cellular infiltration, extracellular matrix deposition, and neovascularization observed with Permacol, its use as a dermal substitute for wound healing has been reduced^7^. However, Permacol has been successfully used for hernia repair, pelvic floor reconstruction, urodynamic stress incontinence and rhinoplasty^6^.

Effective decellularisation methodology is dictated by factors such as cellular density, organization, clinical application and biologic properties of the extracellular matrix (ECM)^8^. ECM is highly conserved amongst species and consists of small proteins, growth factors, collagen and glycosaminoglycans (GAG)^9^ that are essential for providing the cell-to-matrix and cell-to-cell contacts, creating an ideal environment for new tissue growth or restoring homeostasis. Molecules present in the ECM signal cells directly or indirectly to trigger biological events and help orchestrate a sequence of events to promote proliferation and differentiation of the host derived cells^10^.

The mechanisms of ECM scaffold-mediated constructive remodeling are not fully understood. Cell recruitment and the release of bioactive peptides by protease-mediated ECM degradation are thought to play a role in the constructive remodeling process and in wound healing^11^.

Wound healing is a complex, dynamic, multicellular process that involves several overlapping stages including inflammation, formation of granulation tissue, re-epithelialization and remodeling^12^. This process is regulated by an equally complex signaling network involving numerous growth factors, chemokines and cytokines^13^,^14^. Transforming growth factor (TGF)-β, platelet-derived growth factor (PDGF), basic fibroblast growth factor (bFGF), epidermal growth factor (EGF), vascular endothelial growth factor (VEGF) and the interleukin (IL) family^12^ are of particular interest amongst innumerous proteins present in the wound milieu.

The first phase of wound healing begins with capillary damage and clot formation. It is then followed by a number of events, including the production of pro-inflammatory cytokines and chemokines^15^, recruitment of inflammatory cells such as neutrophils and macrophages^16^, and also fibroblasts and endothelial cells^17^. In the second phase (proliferative phase), the process of neovascularization with delivery of nutrients and oxygen to the wound bed will contribute to fibroblast proliferation^14^. The fibroblasts become activated and, triggered by TGF-β1, acquire a smooth muscle cell-like phenotype called myofibroblasts^17^,^18^. These cells exhibit contractile properties, due to the expression of alpha - smooth muscle actin (α-SMA) in microfilament bundles, playing a major role in the contraction and maturation of granulation tissue^19^. During the remodeling phase, the number of vascular cells and myofibroblasts are dramatically reduced by apoptosis^20^. In this scenario, the key orchestrators and effectors of the remodeling progression - macrophages - are known to alter their function and phenotype to meet the needs of the healing tissue^21^.

During the proliferative phase, macrophages assume a wide spectrum of different functional phenotypes that can influence repair^22^. The exact mechanisms are still poorly understood, but it has been thought that the macrophage phenotype switch during tissue remodeling may be driven, in part, by phagocytosis of tissue debris and apoptotic cells^21^. It has also been observed that transition between the pro-inflammatory (M1) to the anti-inflammatory (M2) phenotype is followed by a decrease in VEGF and pro-inflammatory cytokine production (IL-1, IL-6), with an increase in IL-10 secretion^23^,^24^ and TGF-β^25^.

In the context of regenerative medicine, the effect of macrophage phenotypes upon biologic scaffold remodeling has already been shown^26^. Our study however discuss the pleiotropic effect of macrophages upon different cell types and molecular responses evaluated by the host tissue reaction to two non-crosslinked skin scaffolds tailored by different decellularisation processes and one commercially available crosslinked dermal matrix well established for soft-tissue repair.

## Results

### Evaluation of scaffolds

**Figure 1:**
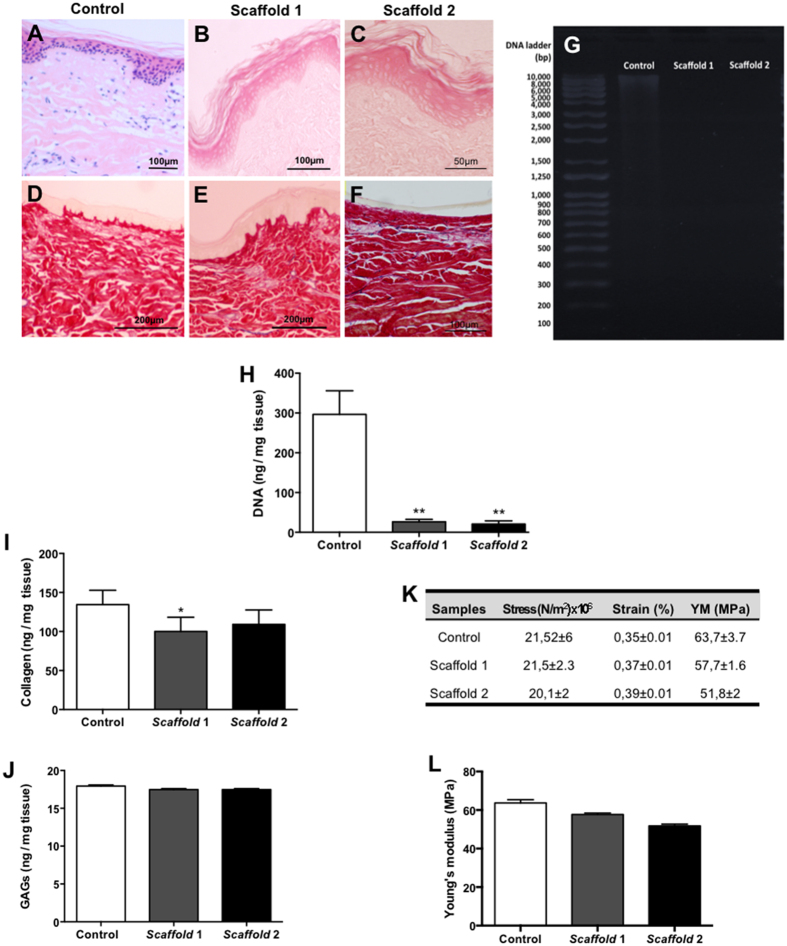
The acellular skin scaffolds produced by an enzymatic decellularisation method show preserved ECM components. Light microscopy analysis showing native porcine dermal matrix (**A**) and scaffolds 1 (**B**) and 2 (**C**). The collagen fibers of the scaffolds showed the classical collagen fiber architecture and distribution in both scaffolds (**E**,**F**), when compared to the non-decellularised control skin (**D**). Analysis of DNA naturally occurs in the normal skin and be absent in the scaffolds 1 and 2 (**G**,**H**). Quantitative analysis of collagen type 1 (**I**) and glycosaminoglycans (GAGs) (**J**). Tensile strength test related to the mechanical properties of scaffolds (1 and 2) and control groups (**K**,**B**). Stain: Haematoxylin and Eosin (**A**–**C**); Picrosirius-haematoxylin and Miller Elastin (**D**–**F**). Data indicate the mean ± S.E.M. of DNA and collagen ng per mg of tissue (n = 5 samples/group). *p < 0.05 versus control; **p < 0.01 versus control.

Both decellularisation methods (described in Table 1) used to produce the acellular matrices proved to be time (<3 days) and cost-efficient, producing ‘off-the-shelf’ skin scaffolds, which is very desirable in regenerative medicine. Following decellularisation, samples were assessed histologically using HE-stained sections. Figure 1A shows the histological structure of native porcine skin, whereas the scaffold revealed the complete removal of cells and nuclear material from the matrix (Fig. 1B,C). The collagen fibers of the scaffolds showed the classical collagen fiber architecture and distribution (Fig. 1E,F), when compared to the non-decellularised control skin (Fig. 1D). Furthermore, DNA quantification showed that approximately 90% of nuclear material was depleted after both decellularisation protocols, with no visible DNA on the electrophoresis gel following staining by ethidium bromide (Fig. 1G,H).

The ECM components of the bioengineered skin scaffolds were assessed by quantification of collagen and glycosaminoglycans (GAGs). Collagen content showed to be significantly decreased in the scaffolds 1 (100 ± 8 μg collagen/mg tissue) when compared to control skin (134 ± 8 μg collagen/mg tissue). This decrease was not significant in the scaffold 2 (109 ± 8 μg collagen/mg tissue) (Fig. 1I). The amount of GAGs (Fig. 1J) however was well-preserved after decellularisation (scaffold 1 = 16.04 ± 0.65 μg; scaffold 2 = 16 ± 0.65; control = 16.52 ± 0.68 GAGs/mg tissue).

The mechanical properties were evaluated in all the groups by tensile strength test, measured as the maximum stress that each sample could be stretched to withstand before breaking. The results showed that both decellularisation processes did not change the mechanical properties of the scaffolds when compared to the control group (Fig. 1K,L).

### Histological analysis of acellular matrix and host tissues: biocompatibility

The porcine skin scaffolds (1 and 2) and Permacol were implanted subcutaneously in rats and its biocompatibility was assessed on the 3°, 14° and 21° days.

**Figure 2:**
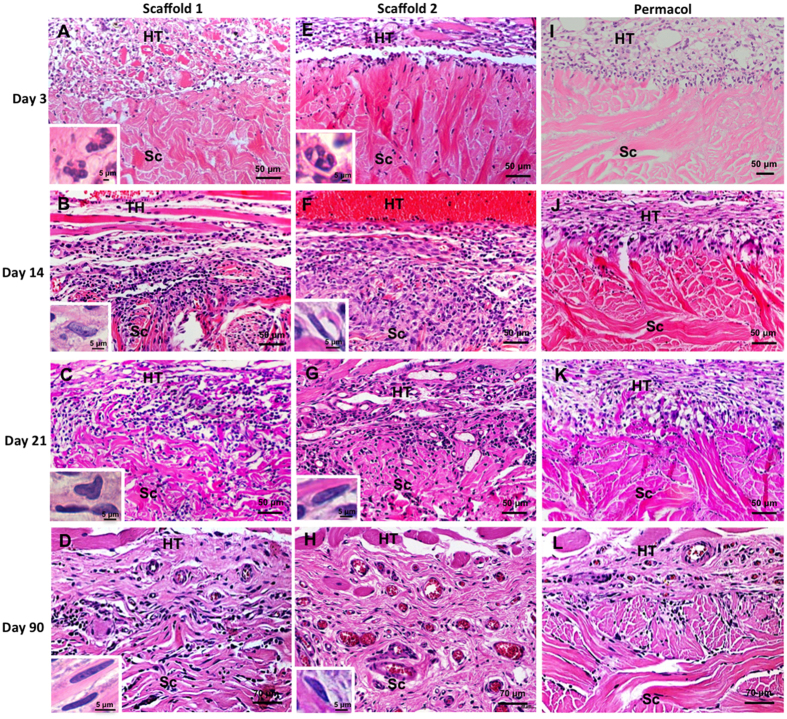
Histopathological evaluation performed after acellular matrix scaffolds implantation. Scaffolds without cross-linked 1 (**A**–**D**) and 2 (**E**–**H**), and Permacol with cross-linked (**I**–**L**) after days 3, 14, 21 and 90 of the surgical procedure. At day 3 of implantation, scaffolds 1 and 2 were surrounded by an accumulation of PMN cells (**A**,**E** - insets) and after day 14 were mostly mononuclear cells and myofibroblasts/fibroblasts (**B**–**D**,**F**–**H** - insets). Histologic appearance of the Permacol showed a mild reaction of the host tissue and limited cell infiltration (**I**–**L**). Host tissue (HT). Implant (Sc). Staining: Haematoxylin and Eosin.

Histological analysis indicated that the implants were biocompatible, with no evidence of rejection or fibrosis. At day 3 of implantation, both scaffolds 1 and 2 were surrounded by an accumulation of polymorphonuclear (PMN) cells (Fig. 2A,E - insets); and from day 14, cell population was replaced by mononuclear cells, most likely macrophages and fibroblasts/ myofibroblasts distributed throughout both scaffolds (Fig. 2B–D,F–H - insets). Histologic appearance of the Permacol (used as surgical control) at day 3 after surgery showed a mild reaction of the host tissue and the cell infiltration was limited almost exclusively to the edge of the matrix, and slowly increased up to 90 days (Fig. 2I–L).

**Figure 3:**
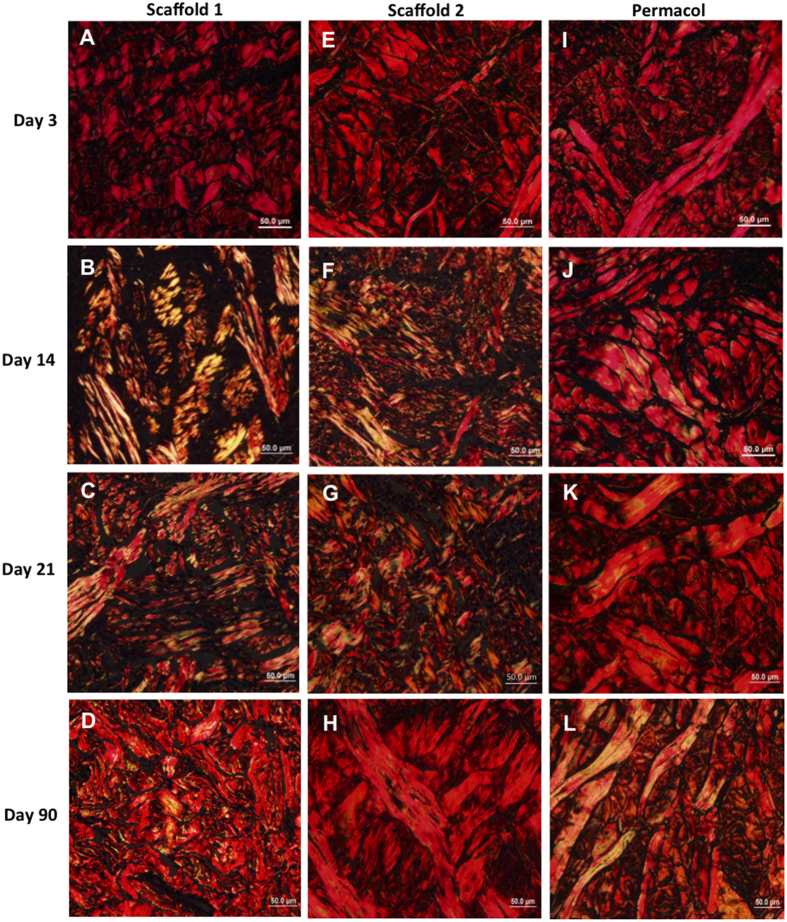
Implants under polarized light showing naturally birefringent collagen fibers. Analyses of collagen fibers in the scaffolds 1 (**A**–**D**) and 2 (**E**–**H**), and Permacol (**I**–**L**) after days 3, 14, 21 and 90 of the surgical procedure. At day 3 post-implantation, all implants displayed a uniform distribution of collagen fibers (**A**,**E**,**I**). In contrast, histologic appearance shows a disorganized connective tissue from day 14 to 21 (**B**,**C**,**F**,**G**) and a significantly increased collagen deposition at day 90 (**D**,**H**). The Permacol matrices were still identifiable throughout the study (**I**–**L**). Staining: picrosirius-haematoxylin.

The collagen fibers of the scaffolds and Permacol were observed by picrosirius-haematoxylin polarization technique. At day 3 post-implantation, all implants displayed a uniform distribution of collagen fibers, which showed to be strongly birefringent under polarized light (Fig. 3A,E,I). In contrast, there was a significant decrease in collagen at the site of scaffold implantation at day 14 (Fig. 3B,F) when compared to day 3 (Fig. 3A,E), showing degradation and rearrangement of the matrices. Histologic appearance of a disorganized connective tissue from day 14 to 21 (Fig. 3B,C,F,G) and a significantly increased collagen deposition at day 90 (Fig. 3D,H), suggested that remodeling of connective tissue was taking place. The implanted Permacol matrices, however, were still identifiable throughout the study (Fig. 3I–L).

### Cellular changes after scaffolds implantation: immunohistochemistry

**Figure 4:**
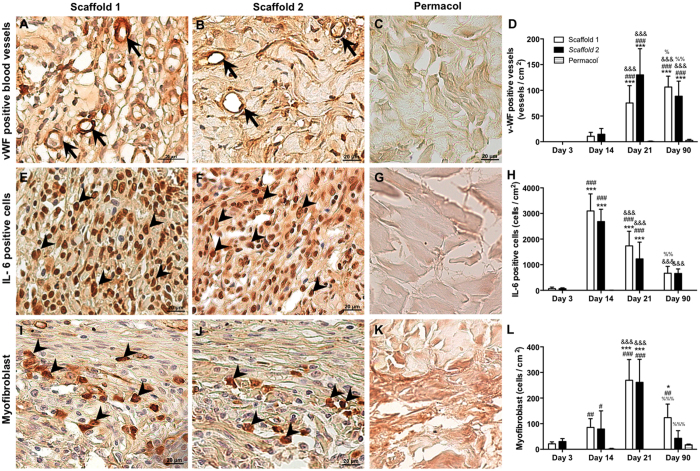
Analysis of angiogenesis and cell populations during the remodeling process. Immunohistochemical (day 14 representative image) and quantitative analysis of von Willebrand factor positive blood vessels (arrows) (**A**–**D**), IL-6 positive cells (arrows head) (**E**–**H**), and myofibroblasts (arrows head) (**I**–**L**) within the scaffolds (1 and 2) and Permacol at 3, 14, 21 and 90 days after implantation. Counterstaining: haematoxylin. Data indicate the mean ± S.E.M. of von Willebrand factor positive blood vessels and cells per cm^2^ (n = 5 animals/group). ^#^p < 0.05 versus respective Permacol; ^##^p < 0.01 versus respective Permacol; ^###^p < 0.001 versus respective Permacol; *p < 0.05 versus day 3; ***p < 0.001 versus day 3; ^&^p < 0.05 versus day 14; ^&&&^p < 0.001 versus day 14; ^%^p < 0.05 versus day 21; ^%%^p < 0.01 versus day 21; ^%%%^p < 0.001 versus day 21.

The angiogenesis was analyzed by immune staining of the multimeric glycoprotein Von Willebrand factor (vWF) (Fig. 4A–C) in all scaffolds. The newly formed vessels were seen from day 14, with a significant increase at day 21 (Fig. 4D). While in Permacol there was no increase to over time (day 3 = 0; day 14 = 0; day 21 = 0.6 ± 0.2; day 90 = 2.5 ± 0.6).

Immunohistological methods were used to identify different cell populations during the remodeling process. IL-6 positive cells were quantified (Fig. 4E–G), and showed to be significantly increased in both scaffolds after day 14 (scaffold 1 = 3099 ± 332; scaffold 2 = 2686 ± 238; p < 0.01), with progressive decrease in subsequent periods, when compared to Permacol (day 3 = 0; day 14 = 6 ± 2; day 21 = 9 ± 4; day 90 = 2 ± 1) (Fig. 4H).

Myofibroblasts, that are primarily responsible for wound contraction, have been identified by staining the cytoplasmic protein alpha-smooth muscle actin (α-SMA) (Fig. 4I–K), which is commonly used as a marker of myofibroblast differentiation. A marked increase of the α-SMA-positive cells was observed on day 21 (scaffold 1 = 269 ± 29; scaffold 2 = 261 ± 29; p < 0.01), followed by an expressive reduction on day 90 (Fig. 4L).

**Figure 5:**
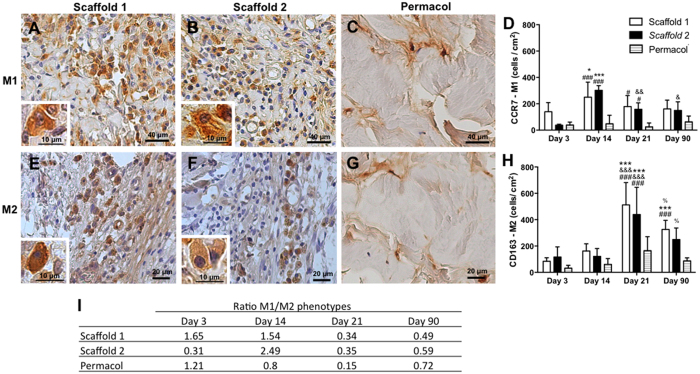
Macrophages phenotypes modulation in scaffolds (1 and 2) and Permacol after implantation. Immunohistochemical (day 14 representative image) and quantitative analysis of CCR7 (**A**–**D**) and CD163 (**E**–**H**) positive macrophages at 3, 14, 21 and 90 days. The insets show higher magnifications of the macrophages. Counterstaining: haematoxylin. Data indicate the mean ± S.E.M. cells per cm^2^ (n = 5 animals/group). ^#^p < 0.05 versus respective Permacol; ^###^p < 0.001 versus respective Permacol; *p < 0.05 versus day 3; ***p < 0.001 versus day 3; ^&^p < 0.05 versus day 14; ^&&^p < 0.01 versus day 14; ^&&&^p < 0.001 versus day 14; ^%^p < 0.05 versus day 21.

To identify macrophage populations, surface markers CCR7 (M1 macrophages) and CD163 (M2 macrophages) were used. Scaffolds 1 and 2 elicited predominately a CCR7+ response at day 14 (scaffold 1 = 217 ± 24; scaffold 2 = 218 ± 36; p < 0.01) (Fig. 5A,B,D), while CD163+ profile showed to be significantly increased only after day 21 (scaffold 1 = 512 ± 62; scaffold 2 = 439 ± 75; p < 0.001) (Fig. 5E,F,H), and persisted until day 90 in both scaffolds 1 and 2 (Fig. 5D,H). Permacol, in turn, showed the lowest population of mononuclear cells characterized by a similar number of CCR7+ and CD163+ macrophages (Fig. 5C,D,G,H).

The ratio between M1/M2 phenotypes was calculated and showed in the Fig. 5I. Although the initial response to the scaffolds implantation showed a predominant inflammatory reaction (with values consistently above 1.0, which indicates a M1 macrophage response), the host tissue reaction seemed to equalize and displayed a prominent M2-profile thereafter.

### Molecular changes after scaffolds implantation: qRT-PCR

**Figure 6:**
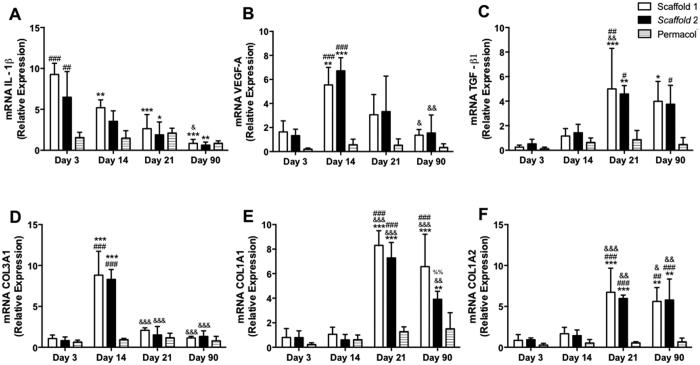
Gene expression of trophic factors involved in tissue remodeling. Scaffolds (1 and 2) and Permacol at 3, 14, 21 and 90 days were employed in the mRNA extraction with Trizol^®^ reagent. The PCR reaction was performed with specific primers for the detection of mRNA levels of IL-1β (**A**), VEGF-A (**B**), TGF-β1 (**C**), COL3A1 (**D**), COL1A1 (**E**) and COL1A2 (**F**). Data indicate the mean ± S.E.M. of mRNA relative expression (n = 5 animals/group). ^#^p < 0.05 versus respective Permacol; ^##^p < 0.01 versus respective Permacol; ^###^p < 0.001 versus respective Permacol; *p < 0.05 versus day 3; **p < 0.01 versus day 3; ***p < 0.001 versus day 3; ^&^p < 0.05 versus day 14; ^&&^p < 0.01 versus day 14; ^&&&^p < 0.001 versus day 14; ^%%^p < 0.01 versus day 21.

Gene modulation after implantation of scaffolds (1 and 2) and Permacol was assessed by fluorogenic qRT-PCR-based (TaqMan) assay. Scaffolds 1 and 2 showed to trigger a strong inflammatory response seen by a substantial increase on the IL-1β mRNA expression, which started at day 3 (scaffold 1 = 9.2 ± 1 and scaffold 2 = 6.5 ± 1.9-fold increase; p < 0.001) and ceasing only around day 21 (scaffold 1 = 2 ± 1 and scaffold 2 = 1.6 ± 0.9 - fold increase) (Fig. 6A). Vascular endothelial growth factor A (VEGF-A) mRNA expression displays a bell-shaped curve with a significant augmented expression on days 14 (scaffold 1 = 5.5 ± 0.7 and scaffold 2 = 6.7 ± 0.5 - fold increase; p < 0.01) (Fig. 6B). The expression of the transforming growth factor-beta 1 (TGF-β1) however showed to be delayed, revealing a significant increase only at day 21 after implantation (Fig. 6C).

The marker genes for collagen remodeling COL1A1, COL1A2 and COL3A1 displayed an antagonist behavior. A marked increase on the collagen 3 (Fig. 6D) was seen at day 14 with a substantial decrease on day 21, when collagen 1 (COL1A1 and COL1A2; Fig. 6E,F) displayed its maximum expression (~8-fold increase in COL1A1 and 7-fold increase in COL1A2).

Permacol did not stimulate significantly any of those classical markers of wound healing during the studied time points (Fig. 6A–F).

## Discussion

Biologic scaffolds are commonly used to promote repair and restoration of functional tissues^27^. Although most, if not all of these scaffolds are composed of ECM or components of ECM, the clinical and tissue remodeling outcomes may differ greatly^26^. The bioactivity of scaffolds can be altered depending on the type of decellularisation reagents and processes used, eliciting distinct host-tissue morphologic and molecular responses. Models of wound healing in rodents can provide reliable and reproducible information on the response of wound contaction to the experimental therapy^28^. Given the remarkable rate of advancement in wound healing in animal species, research may be able to develop more incisive models of the healing process and its key steps^28^,^29^.

In this study, we used a rat model to evaluate the cellular and molecular response to implantation of two decellularised non-crosslinked porcine skin scaffolds in comparison to a commercially available crosslinked dermal scaffold (Permacol). Permacol has been used for years as a surgical implant, providing durability and strength for ventral hernia repair and abdominal wall reconstruction^30^,^31^.

The surgical sites at which the scaffolds were placed showed a dense infiltration of polymorphonuclear (PMN) and some mononuclear cells, which persisted up to day 21. A possible systemic reaction was discarded by measuring leukocytes and inflammatory mediators in the blood of the animals after implantation (Supplementary Figure 1).

Both scaffolds displayed an uniform collagen remodeling when observed under polarized light (picrosirius-haematoxylin staining), but not the HDMI-crosslinked porcine dermal scaffold Permacol. Cross-linking agents, such as 1,6-hexamethylene diisocyanate (HDMI), are included in the processing of biologic materials to impart strength and to slow the degradation rate once implanted. Permacol being a crosslinked matrix could be distinctly seen with little reorganization of host connective tissue, and with insignificant infiltration of cells (IL-6-positive, macrophages and myofibroblasts) and blood vessels up to day 90. Our results are in agreement with other studies demonstrating that treatment with any crosslinking reagents can change the collagen architecture at a molecular level^32^. These molecular alterations impact directly on cell surface interactions^33^, and can change the quantity and identity of secreted pro-inflammatory cytokines and chemokinesm^34^, the gene expression pattern^35^, and downstream remodeling events^36^,^37^. Kulig and collaborators (2013) showed that various non-crosslinked scaffolds commercially available, such as Alloderm, Gelfoam and Stattice, have lower cell migration and proliferation when compared to Permacol, despite of the latter being a crosslinked matrix. These results suggest that biologic and structural properties of both type of scaffolds need to be carefully studied to assess new products and its uses in this promising class of biomaterials^38^.

The non-crosslinked scaffolds 1 and 2 showed a similar degree of biocompatibility, with a pronounced cell infiltration from day 3 and much higher biodegradability when compared to Permacol. The angiogenic response was initiated on day 14, and at day 21 the number of newly formed vessels, visualized by immunostaining of von Willebrand factor (vWF), had significantly increased.

Although the non-crosslinked scaffolds 1 and 2 showed a similar degree of biocompatibility, a slightly delayed integration with host tissue, cellular infiltration and biodegradability was seen on scaffold 2 when compared to scaffold 1. Although these differences were not significant, they may be attributed to the loss of the glycosaminoglycans (GAGs) content evidenced in the scaffold 1, probably caused by the reagents used in the decellularisation process. It has been shown that Triton-X, especially when combined with other detergents or enzymatic digestion (included in the manufacturing of scaffold 1), can lead to a certain loss on ECM components, resulting in early tissue degradation^33^.

The long-term remodeling outcome differs greatly amongst scaffold materials^26^. The maintenance of the ECM structure and biomechanical properties of the non-crosslinked scaffolds supported a satisfactory tissue remodeling when compared to the cross-linked Permacol. Nevertheless, depending on the particular application, it may be advantageous to use crosslinked scaffolds to limit the degree of subsequent scaffold degradation, which is crucial when scaffolds are intended to be used as permanent and non-absorbable matrices.

The temporal sequence of remodeling events of extracellular matrix devices, including the speed of scaffold degradation and the extent of new-tissue deposition by the host, may be predictive of the course of wound healing and outcome of the procedure^27^. In order for wounds to heal timely and properly, there must be a fine balance of interaction between various cell types, cytokines, growth factors, proteases and ECM components. Coordinated efforts of cells including leukocytes^16^, platelets, fibroblasts, endothelial cells^14^ and macrophages^39^,^40^ occur in order to promote formation of new tissue and wound closure.

In commonly used experimental models, the inflammatory phase is generally limited to the first few days after injury. Within hours after acute injury, inflammatory cells begin to accumulate at the affected site^41^. In common experimental models using healthy young male mice, neutrophil accumulation peaks around day 1 and returns to uninjured levels by days 5 to 10 after skin wounding^42^. The proliferative phase begins within days after injury and peaks within 1 week after injury; and the remodeling phase begins as proliferation starts to subside, and can last for months or more^21^.

Our results however indicated that, after skin scaffolds implantation, the remodeling process was somehow delayed. This could be attributed to an increased initial inflammatory response, which is clearly seen with an extensive infiltration of PMN cells and a concurrent amplified IL-1β mRNA expression, which showed a return to baseline conditions only after 14 days. This could have been caused by residues from reagents or components of the implanted scaffolds that could have directly or indirectly affected the cellular events. Collagen-based matrices have been used in several models for different medical applications^43^, but despite the desired effect, these materials can cause a relatively severe local inflammatory reaction after implantation, depending on the structure and functional characteristics of the material^44^. On the other hand, at a later stage our results did not show any complications related to the delayed proliferative phase, such as persistent inflammation or low-grade chronic inflammation, poor tissue organization, presence of foreign-body giant cells or fibrous encapsulation as shown by other authors^45^,^46^.

The different phases of wound healing is strictly regulated by multiple growth factors and cytokines^14^ that regulate migration, proliferation, and differentiation of cells^47^. Of particular interest, IL-6 has been shown to be important in the remodeling response^12^. Our results showed that IL-6 positive cells were increased at day 14 and successively decreased thereafter. Those positive cells have the morphology of large mononuclear cells and we suggest that they may be monocytes that migrated into the tissue and started the transition into macrophage phenotypes. Sequential activation of classic, pro-inflammatory, M1 macrophages and alternatively activated or anti-inflammatory M2a, M2b, and M2c macrophages occurs during normal healing and facilitates transitions through the inflammatory, proliferative, and remodeling phases of repair^22^. Although morphologically indistinguishable using routine methods of examination, macrophages phenotypes can be identified and distinguished according to their cell surface markers and their cytokine and gene expression profiles^26^,^48^.

M1 phenotype (CCR7+ cells) accumulation occurred in our study on day 14. This is divergent with other studies showing that the M1 peak is somewhat earlier, around days 3 to 7, and declines significantly by days 10 to 14, although low levels of macrophage accumulation may persist for weeks^41^,^49^,^50^.

Depletion of macrophages during the inflammatory phase reduces granulation tissue formation and cell proliferation in mouse skin wounds^39^ and prevents formation of new myofibers in injured muscle^51^.

Although the exact mechanisms by which macrophages regulate these processes remain to be determined, cytokine production may be one mechanism by which macrophages of the inflammatory phase initiate transition into the proliferative phase of healing^26^.

Interestingly, in our study we showed a timely gene expression shift marking this phenotype transition, which is in agreement with other authors^24^,^52^,^53^. mRNA expression of the vascular growth factor (VEGF-A) which showed to be strongly up-regulated until day 14, started subsiding as the transforming growth factor beta 1 (TGF-β1) gene expression began increasing. This was mirrored by an augmented number of CCR7+ (M1 macrophages) at day 14 and a low number of CD163+ cells (M2 macrophages) observed at the implantation site. After day 14, however, the M1/M2 ratio indicated a predominant M2 response in both tested scaffolds.

Many growth factors and cytokines secreted by macrophages have pleiotropic influence on cell proliferation, angiogenesis, and extracellular matrix synthesis. To mention few, VEGF-A was shown to be strongly induced after cutaneous injury, with keratinocytes and macrophages being the major producers^53^. Its major function in wound healing is to promote cell differentiation and angiogenesis^39^,^54^. IL-6, also secreted by macrophages, initiates the healing response^55^ and induces regulation of proliferation of myofibroblasts^24^,^56^. And also, not less importantly, the TGF-β1 that is known as the signature mediator of myofibroblasts differentiation^57^.

For successful wound healing, it is important that inflammation, angiogenesis and reepithelialization processes take place^14^,^58^,^59^. Non-crosslinked porcine skin scaffolds may allow the sequence of wound healing events to take place leading to an enhancing successful reepithelialization in skin wound models. According to Hoganson and collaborators (2010) the retention of a wide array of ECM proteins and cytokines makes those non-crosslinked matrices very promising assets showing potential advantages in some surgical applications. The retention of angiogenic growth factors and TGF-β suggests that those non-crosslinked matrices may support excellent fibroblast infiltration and collagen production when used for hernia repair and other surgical reconstruction applications^60^.

In our study, the mRNA VEGF-A/TGF-β1 switch seemed not only to hallmark the M1/M2 transition but also to overlap actions on myofibroblast differentiation, neovascularization and synthesis of different types of collagen. It has been shown that collagen deposition in skin wounds appears to be controlled by monocytes/macrophages of the inflammatory phase, rather than macrophages of later phases^39^,^61^. Although pronounced transcription of COL1α1 and COL1α2 can be up-regulated as early as 2 to 3 days after injury^42^,^61^, our results showed that those genes were up-regulated at day 21. Nonetheless, studies show that both early mRNA and late protein levels of collagen, when reduced by depletion of monocytes/macrophages during the inflammatory phase, seem to result in delayed healing but reduced scar formation^52^.

One of the characteristics of wound remodeling is the change of extracellular matrix composition. We therefore assessed the expression of the collagen marker genes COL1A1, COL1A2 and COL3A1 in response to skin scaffolds implantation and the results showed that the transition from collagen type III to I occurred around day 21, when we also see an increased number of myofibroblasts in the site of implantation.

Myofibroblasts are the predominant mediator of the contractile process because of their ability to extend and retract^14^. During granulation tissue formation, fibroblasts are gradually modulated into myofibroblasts, which are characterized by increased expression of smooth muscle differentiation markers such as alpha–smooth muscle actin (α-SMA), smooth muscle myosin, and desmin starting after the first week, and reaching a peak after day 15^62^.

Although there are many interconnected cells and molecular events to be further explored in more detail, our results suggest that modulation of myofibroblasts and ECM remodeling in the implantation site seems to be timely associated with the M2 response.

In the context of biomaterials, the large spectrum of events that underlines tissue homeostasis showed to be regarded, at least in part, to the well-known heterogeneity of macrophage phenotypes^26^,^39^. The relatively late responsiveness of those cells after non-crosslinked biological scaffolds implantation, in our study, seems to intimately affect various other cell responses; and this range of mutually reinforcing actions was predictive of a positive tissue remodeling result.

The classical view of wound healing shows a timed sequence of cellular events directed largely by cytokines and growth factors that predicate the functional outcome of the newly formed tissue. Our study suggested that non-crosslinked biologic scaffold implantation somehow delayed the sequence of cell responsiveness and underlying molecular events involved in tissue repair. The late macrophage response seemed to modulate a network of cellular events and molecular feedback, promoting a favorable remodeling response that is essential for long-standing success of the implant. Our data showed optimum biological properties of the non-crosslinked scaffolds, which may be useful tools as skin substitutes in reconstructive surgeries, and can also reduce morbidity, improving functional outcomes in clinical settings^6^.

## Materials and Methods

### Dermis harvesting and scaffold production

Fresh porcine skin was obtained from Large-White/Landrace crossbreed pigs (6 months old) after euthanasia. Skin was cleansed with soap, shaved and washed with water and iodine based solution (10% w/w Cutaneous Solution - Iodinated Povidone, Videne, Garforth, UK). The intact skin (epidermis and dermis) was dissected from the animal’s flank, washed in sterile phosphate buffered saline (PBS, Sigma-Aldrich, Dorset, UK) with an antibiotic/ antimycotic solution (AA; Sigma-Aldrich, Dorset, UK) and stored in sterile plastic bags at −20 °C for 24–48 h. The skin samples were defrosted, cut (2 × 2 cm), and allocated randomly into 2 groups for the production of decellularised scaffolds 1 and 2 (Table 1). This study was approved by Ethics Committee in Animal Experimentation of São Paulo State University (UNESP) (protocol no. 062/2012 – CEUA) and was performed according to the regulatory guidelines of the UK Home Office.

### Histological analysis of scaffolds

Samples from scaffolds (1 and 2) and Permacol (TSL, Hampshire, UK), a commercially available cross-linked porcine acellular dermis used as surgical control, were fixed for 24 h in 10% NBF solution. They were washed, dehydration in graded alcohol, embedding in paraffin wax and sectioned at 5 μm. Separate sections were stained with Haematoxylin and Eosin (HE) and Picrosirius-haematoxylin (picrosirius-haematoxylin stained sections were analyzed under polarized light to assess degradation and tissue remodeling at the implantation site).

### DNA quantification and gel electrophoresis of scaffolds

Samples were cut into small pieces, weighed and stored aseptically in DNA-free tubes. DNA was extracted following manufacturer’s instructions of GenElute mammalian genomic DNA miniprep kit (Sigma-Aldrich, Dorset, UK). Total DNA was quantitated by measuring the absorbance in a nano-drop spectrophotometer (NanoDrop ND1000, Thermo Scientific, Wilmington, USA), and the absolute amount of DNA per milligram of tissue was calculated.

The size, quality and purity of the extracted DNA were determined by electrophoresis. A 1.2% agarose (Agarose Type I, low EEO, Sigma-Aldrich, Dorset, UK) gel with 1x Tris-borate-ethylenediaminetetraacetic acid (TBE - Bio Reagent, 10x, Sigma-Aldrich, Dorset, UK) running buffer was run at 4 to 5 V/cm between the electrodes. Equal volumes of DNA (5 μL) and 1 μL of loading buffer (5x DNA loading buffer, Yorkshire Bioscience Ltd., York, UK) were loaded into each well. Visualization was achieved by staining with 1% of ethidium bromide and DNA samples were measured via ultraviolet transllumination against a 1-kb DNA ladder (Q-Step 4 quantitative DNA ladder, Yorkshire Bioscience Ltd., York, UK).

### Glycosaminoglycan (GAG) quantification of scaffolds

To quantify GAG content on both scaffolds and control (non-decellularised skin), the Blyscan GAG assay kit (Biocolor, Carrickfergus, Northern Ireland) was used^33^. The absorbance was measured using a plate reader (Versamax, Molecular Devices LLC, USA) at 656 nm and the absolute GAG content was calculated per milligram of tissue.

### Collagen quantification of scaffolds

Collagen was quantified using Sircol collagen assay kit (Biocolor, Carrickfergus, Northern Ireland)^33^. Extracts were placed in a 96-well plate in triplets and spectrophotometric readings were taken at 555 nm on a microplate reader (Versamax, Molecular Devices LLC, USA). Absolute values were attained with a standard curve composed of type I bovine skin collagen solution (0.5 mg/mL) in the range of 5–100 μg per 0.1 mL. Total collagen was normalized per milligram of tissue.

### Biomechanical test of scaffolds

Mechanical properties of non-decellularised skin control and scaffolds 1 and 2 (n = 5) were evaluated by a tensile strength test measured as the maximum stress that each sample could with stand whilst being stretched before reaching breaking point. For each test, specimens were cut into “dumbbell” shapes using a standard mold with 2.5 cm in length and 0.4 mm in width. The tests were performed with the application of uniaxial tension using a tensiometer (InstronInspec 220 Benchtop Portable tester, Instron, Buckinghamshire, UK). Samples were clamped into the holders and loaded at a constant tension rate. The tensile tester recorded the real-time load and elongation to which the tissue was subjected. Parameters such as maximum load (N), testing time (sec) and extension at maximum load (mm) were recorded. Strain was defined as rate of change of sample deformation and calculated as a ratio of the length at the maximum load to the original length; and stress was defined as how much load was applied at each meter squared sample. Young’s modulus (YM; MPa) was calculated by the ratio of stress (N/m^2^) over strain (%).

### Experimental Animals

Male Wistar rats (250–300 g body weight), aged from 6 to 8 weeks old, were randomly distributed into 9 groups (n = 5 per group) and kept in a 12 h light/dark cycle with food and water ad libitum.

### Study Design

Both scaffolds and the control Permacol were cut (1 × 0.3 cm) and kept in saline prior to implantation. The animals were anesthetized with intramuscular administration of ketamine (100 mg/kg) and xylazine (10 mg/kg). The skin was shaved and cleaned using iodine based solution (10% w/w Cutaneous Solution - Iodinated Povidone, Videne, Garforth, UK). Ventral incisions (0.5 cm) were performed just inferior to the costal margin bilaterally. The subcutaneous tissue was dissected with scissors to expose a ‘pocket’ of approximately 1 cm where the scaffolds were placed. The scaffolds were positioned and fixed subcutaneously.

After 3, 14, 21 and 90 days after implantation, the animals (n = 5 for both tested scaffold and Permacol) were weighed and euthanized with an intra-peritoneal injection of sodium pentobarbitone. The scaffolds (Sc) as well as adjacent areas were collected and stored either in -80 °C or neutral buffer formalin (NBF).

### Processing of skin fragments for histological analysis

The samples were processed for histology as described in “Histological analysis of scaffolds” session. Picrosirius-haematoxylin was used to enhance the natural birefringence of collagen and allow quantification of collagen fibers in the implantation site. HE staining was used for general assessment of the host tissue, implant integration, as well as general tissue reaction. An average of 5 fields were randomly selected and analyzed by two independent individuals using the commercial software Image-Pro Plus 6.0.

### Immunohistochemical analysis

Sequential fragments of each specimen were cut at 4 μm intervals. Representative sections of each sample were prepared for immunohistochemical staining by deparaffinization with xylene and rehydration through a graded ethanol series. Sections were submitted to an antigen retrieval step at 96 °C for 30 min, according to Table 2. The endogenous peroxide activity was blocked with 3% hydrogen peroxide for 30 min followed by a blocked with 10% BSA/PBS and an incubation with primary antibodies. Some sections were incubated with 1% BSA instead of the primary antibody to provide a negative control for the reaction. After washing, the sections were incubated with a secondary biotinylated Ab (Histostatin^®^ Bulk Kit, Invitrogen, Frederick, MD, USA). Positive staining was detected using a peroxidase-conjugated streptavidin complex and the color was developed using the DAB Substrate Kit (Invitrogen, Frederick, MD, USA). Finally, the sections were washed in distilled water, counterstained with haematoxylin. To assess neovascularization, the number of blood vessels stained for Von Willebrand factor (vWB) in the implanted scaffolds quantified and showed as mean ± SEM of the positive vessels per cm^2^ (5 random fields using a high-power objective x40). For all other immune-positive cells, data were expressed as cells per cm^2^ counted in 5 randomly selected fields. All sections were analyzed using an Axioskop 2-Mot Plus Microscope (Carl Zeiss, Jena, GR), and the AxioVision software for quantitative analysis.

The details of antigen retrieval, washing buffers and primary antibodies dilution are described in the Table 2.

### Quantitative Reverse Transcription Polymerase Chain Reaction (qRT-PCR)

Fluorogenic qRT-PCR-based (TaqMan) assay was used to detect amplification of the target genes. Briefly, total RNA was extracted using a commercially available kit (Qiagen RNeasy Mini Kit; Qiagen, Hilden, Germany), according to the manufacturer’s instructions, with the following modifications to minimize RNA degradation by abundant skin RNAses. Samples were homogenized using bead-beating technology (Precellys, Bertin Technologies, Montigny-le-Bretonneux, France). Proteins potentially interfering with RNA isolation were removed by incubating the homogenate in 590 μL distilled water and 5 μL Proteinase K solution 20 mg/mL (Life Technologies, Paisley, UK) at 55 °C for 10 min then centrifuged at room temperature for 3 min. Supernatants were combined with 0.5 volumes of ethanol (96–100%) into a Rnase-Dnase free tube and RNA was isolated through a RNeasy mini column. The concentration and purity of the RNA was analyzed using the Nandrop ND-1000 (NanoDrop Technologies, Wilmington, DE). All RNA samples were examined for their purity. The absorbance ratio at A260/A280 nm of all samples was ranged from 1.85 to 2.1, indicating all the samples were pure during the RNA extraction procedure.

Complementary DNA (cDNA) was obtained by reverse transcription (RT) of 1 μg of total RNA, with the Superscript III reverse transcriptase system (Invitrogen, Carlsbad, CA, USA) following the manufacturer’s protocol and using oligo(dT)15 as a primer. Real-time PCR was performed with the Eco Real-Time PCR System (Illumina, San Diego, CA, USA). The following amplification profile was used: UDG Incubation 50 °C for 2 min, AmpliTaq Gold 95 °C for 10 min, PCR 40 Cycles - 95 °C for 15 sec and 60 °C for 1 min. For each reaction, a total volume of 20 μL was used, which consisted of 9 μL of diluted cDNA (10 ng/μL of RNA), 10 μL of TaqMan Gene Expression Master Mix (2X) and 1 μL of TaqMan Gene Expression Assay (20X) (Applied Biosystems, CA, USA). Commercially available primers (*IL-1ß*, Rn00580432_m1; *TGF-ß1*, Rn01475963_m1; *VEGF-A*, Rn01511604_m1; *COL1A1*, Rn01463870_g1; *COL1A2*, Rn00584426_m1; *COL3A1,* Rn01437683_m1 - Applied Biosystems, CA, USA) were used to probe for target mRNA.

Previous results showed that *Hprt1* (Hypoxanthine phosphoribosyltransferase 1), *CycA* (Cyclophilin A), *ACTs* (actins) and *Gapdh* (Glyceraldehydes-3-phosphate dehydrogenase) are suitable potential reference genes for rats^63^. We therefore tested 3 of those potential reference genes (*CycA, Actb and Gapdh*) for validation of their gene expression stability and suitability as internal controls according to the requirements of the MIQE Guidelines. Our preliminary tests for the reference genes showed that although some genes that are implicated in basal cell metabolism may not be stable upon different conditions^64^, *Gapdh* amongst the other reference genes displayed the narrowest expression range with cycle threshold values between 23 and 25 (Ct = 24 ± 1.5). This gene had a very stable expression in all the experimental conditions along with comparable expression characteristics to target genes. For this reason the use of *Gapdh* alone as a reference gene shown in our study was sufficient. mRNA was then normalized relative to *Gapdh (GAPDH*, Rn01462662_g1; Applied Biosystems, CA, USA), which was subsequently used to calculate expression levels of the target genes. The comparative Ct method was used to measure the gene transcription in samples^65^. Results were expressed as relative units based on calculation of 2-ΔΔCt, which gives the relative amount of target gene normalized to endogenous control (GAPDH) and to the control (sham-operated) samples with the expression set as 1. Negative controls were either RT without enzyme or PCR without cDNA template.

### Statistical analysis

Results were shown as mean ± SEM. For samples with normal distribution, analysis of variance (ANOVA) was used for repeated measures followed by the Bonferroni test. The Kruskal-Wallis test followed by Dunn’s test was used for samples with non-normal distribution. Statistical significance was defined as p < 0.05.

## Additional Information

**How to cite this article**: Mimura, K. K. O. *et al*. Mechanisms underlying heterologous skin scaffold-mediated tissue remodeling. *Sci. Rep.* **6**, 35074; doi: 10.1038/srep35074 (2016).

## Supplementary Material

Supplementary Information

## Figures and Tables

**Table 1:** Reagents, concentrations, and time used for each decellularisation protocol.

Scaffold 1	Scaffold 2
Freezing (−20 °C, 24 h)	Freezing (−20 °C, 24 h)
Wash buffer^a^ (2 × 12 h)	Distilled H_2_O (6 h)
0.25% TritonX-100 + 0.25% SOC + 0.2 SA in distilled H_2_O (24 h)	Hypertonic solution^c^ (overnight)
Wash buffer (2 × 12 h)	Wash buffer (2 × 6 h)
Incubation buffer^b^ + DNAse/RNAse (24 h)	Hypotonic solution^d^ (overnight)
Wash buffer (2 × 12 h)	Wash buffer (2 × 6 h)
	Incubation buffer + DNAse/RNAse (24 h)
	Wash buffer (2 × 6 h)

^a^Wash buffer: PBS containing 0.05% Tween-20.

^b^Incubation buffer: 400 mM Tris–HCl, 100 mM NaCl, 60 mM MgCl_2_, 10 mM CaCl_2_; pH 7.6.

^c^Hypertonic solution: 1 M NaCl, 10 mM EDTA, 50 mM Tris–HCl.

^d^Hypotonic solution: 5 mM EDTA, 10 mM Tris–HCl.

**Table 2:** Solutions and incubation times for IHC protocols.

	Antigen retrieval	Washing buffer	Primary antibody reference	Primary antibody dilution
vWF	TRIS-EDTA buffer pH 9	PBS	polyclonal rabbit anti-Human Von Willebrand Factor (Dako, Glostrup, Netherland)	1:200 in BSA1%, for 1 h at RT
IL-6	10 mM sodium citrate buffer pH 6	PBS	monoclonal anti-IL-6 (840234, R&D, Minneapolis, USA)	1:100 in BSA1%, for 1 h at RT
Myofibroblast	10 mM sodium citrate buffer pH 6	TBS	Polyclonal rabbit anti-α-SMA (Dako, Glostrup, Netherlands)	1:500 in BSA1%, overnight at 4 °C
CCR7–M1	10 mM sodium citrate buffer pH 6	TBS	Monoclonal rabbit anti- CCR7 (Novus Biologicals, Littleton CO, USA)	1:500 in 1% BSA, for 1 h at RT
CD163–M2	10 mM sodium citrate buffer pH 6	PBS	Monoclonal rabbit anti- CD163 (Hycult Biotech, Uden, Netherlands)	1:500 in 1% BSA, for 1 h at RT
