# Supplementary material for: Mechanisms underlying heterologous skin scaffold-mediated tissue remodeling
Source: Sci Rep. 2016 Oct 11;6:35074. doi: 10.1038/srep35074 (PMC5057165; doi:10.1038/srep35074)
Supplement: Supplementary Information [file srep35074-s1.doc]

**Mechanisms underlying heterologous skin scaffold-mediated tissue remodeling**

**MIMURA, K.K.O.^1^; MORAES, A.R.^2^; MIRANDA, A.C.^2^; GRECO, R.^3^; ANSARI, T.^3^; SIBBONS, P.^3^; GRECO, K.V.^3^; OLIANI, S.M.^1,2,*^**

^1^ From the Post-Graduation in Structural and Functional Biology, Federal University of São Paulo (UNIFESP), São Paulo, SP, 04023-900, Brazil;

^2^ Department of Biology; Instituto de Biociências, Letras e Ciências Exatas; São Paulo State University (UNESP), São José do Rio Preto, SP, 15054-000, Brazil;

^3^ Department of Surgical Research, Northwick Park Institute for Medical Research, University College London (UCL), London, Middlesex, HA1 3UJ, United Kingdom.

**Observations: S.M.O. and K.V.G. share senior authorship of this manuscript.**

* Address correspondence to Prof. Sonia Maria Oliani, Department of Biology; Instituto de Biociências, Letras e Ciências Exatas; São Paulo State University (UNESP), Rua Cristovão Colombo, 2265, São José do Rio Preto, SP, Brazil, 15054-000; Tel: +55 17 32212381, Fax: +55 17 32212390, E-mail: [smoliani@ibilce.unesp.br](mailto:smoliani@ibilce.unesp.br)

### Supplementary Material and Methods

**Quantification of peripheral blood leukocytes**

Under anesthesia, 5 mL of blood was collected by cardiac puncture from each animal prior to excision of scaffolds. Ten microliters were stained with Turk solution (90 μL) and leukocytes were quantified using a Neubauer chamber (Laboroptik GmbH, Friedrichsdorf, Hessen, Germany).

**Expression of proinflammatory cytokines in plasma**

Blood aliquots were centrifuged at 2,500 rpm for 5 min at 4°C to separate the plasma. IL-1β, TNF-α and IL-6 were measured using a biochemical assay detection kit from R&D System Company (Abingdon, UK) according to the manufacturer´s instructions. The concentrations were determined by an optical density reader (Molecular Devices Sunnyvale, CA), and calculated according to a standard curve in pg/mL.

**Supplementary Figure**

**
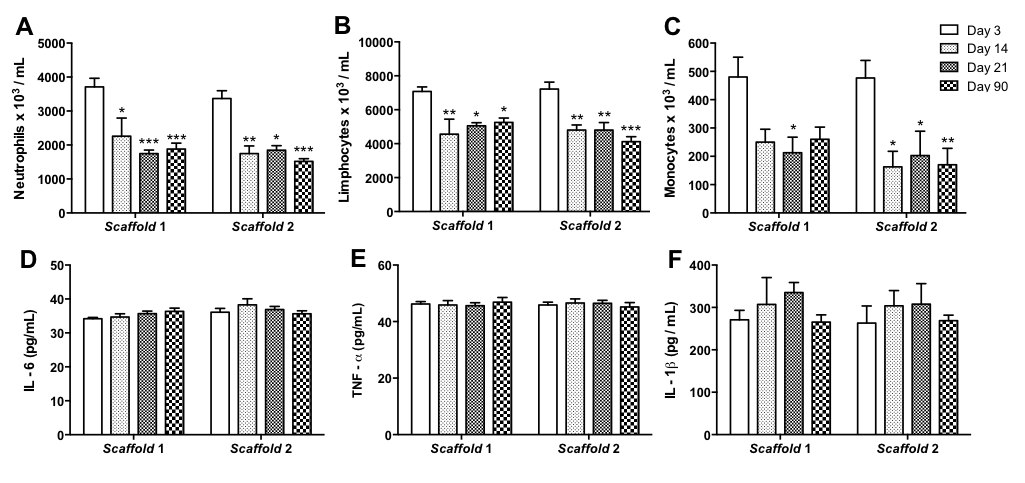
**

**Supplemental Figure 1. Analysis of leukocytes in peripheral blood and proinflammatory mediators in plasma.** Quantitative assessment of neutrophils (A), lymphocytes (B) and monocytes (C) circulating in peripheral blood and dosages of IL-6 (D), TNF-α (E) and IL-1β (F) in periods of 3, 14, 21 and 90 days. Data indicate the mean ± S.E.M. leukocytes x 10^3^ or number of pg of mediators per mL (n = 5 animals / group). * p<0.05 versus 3 days.
